# Validity, reproducibility, and reliability of the Brazilian version of the smartphone addiction scale-short version in children

**DOI:** 10.1590/1984-0462/2025/43/2025126

**Published:** 2025-12-12

**Authors:** Rafael Vieira Martins, Eliane Denise Araújo Bacil, Michael Pereira da Silva, Wagner de Campos

**Affiliations:** a Universidade Federal do Paraná, Curitiba, PR, Brazil.

**Keywords:** Smartphone, Child, Test reproducibility, Test validity, Smartphone, Criança, Reprodutibilidade dos testes, Validade dos testes

## Abstract

**Objective:**

The aim of the study was to assess the validity, reproducibility, and reliability of the Brazilian version of the smartphone addiction scale in children.

**Methods:**

The sample included 194 children (105 girls), aged 8–10 years, enrolled in the public school system of the city of Curitiba (PR). A self-reported questionnaire assessed sociodemographic data (sex, age, type of housing, type of residence, number of siblings, and father's and mother's education). The ABEP (Associação Brasileira de Empresas de Pesquisa) questionnaire assessed economic class, and the Smartphone Addiction Scale-Short Version assessed problematic smartphone use (PSU). Factor analysis was performed to analyze the validity of the PSU scale; the intraclass correlation coefficient (ICC) was used to assess the reproducibility of the instrument; and reliability was measured by internal consistency using Cronbach's alpha (α). All analyses were performed using SPSS (Statistical Package for the Social Sciences) version 21.0 software, adopting a significance level of p<0.05.

**Results:**

Factor analysis resulted in three factors that explained 50.2% of the total variance. The ICC ranged from 0.34 to 0.81, with a value of 0.85 as the overall score. Cronbach's α ranged from 0.35 to 0.81, with an overall score of 0.87.

**Conclusions:**

The scale showed adequate factorial validity and acceptable consistency and reproducibility. The use of this scale is recommended in studies conducted with children aged 8–10 years.

## RESUMO

**Objetivo:**

Avaliar a validade, reprodutibilidade e confiabilidade da versão brasileira da escala de dependência do uso de *smartphone* em crianças.

**Métodos:**

A amostra incluiu 194 crianças (105 meninas), de oito a dez anos de idade, matriculadas na rede pública de ensino da cidade de Curitiba (PR). Um questionário autorrelatado avaliou dados sociodemográficos (sexo, idade, tipo de moradia, tipo de residência, número de irmãos, escolaridade do pai e da mãe). O questionário da (Associação Brasileira de Empresas de Pesquisa) avaliou classe econômica e o Smartphone Addiction Scale-Short Version (SAS-SV) avaliou o uso problemático de smartphone (UPS). Para a análise de validade da escala de UPS foi realizada análise fatorial, para a reprodutibilidade do instrumento foi utilizado o coeficiente de correlação intraclasse (CCI) e a confiabilidade foi medida pela consistência interna com o alfa (α) de Cronbach. Todas as análises foram realizadas por meio do *software* Statistical Package for the Social Sciences (SPSS) versão 21.0, adotando-se nível de significância de p<0.05.

**Resultados:**

A análise fatorial resultou em três fatores, que explicaram 50,2% da variância total. O coeficiente de correlação intraclasse variou entre 0,34 e 0,81, tendo o valor de 0,85 como escore geral. O α de Cronbach variou de 0,35 a 0,81, e o escore geral foi 0,87.

**Conclusões:**

A escala apresentou validade fatorial adequada e consistência e reprodutibilidade aceitáveis. A utilização da presente escala é recomendada em estudos realizados com crianças de oito a dez anos de idade.

## INTRODUCTION

The use of electronic devices, such as smartphones, has grown in recent years throughout the world.^1^ The ease of connecting to the internet, becoming practically like a handheld computer, has made the smartphone the most widely used mobile device globally, as its use helps people to work, study, socialize, and enjoy leisure time.^2-4^

Although there is an understanding of the daily conveniences of smartphone use, prolonged exposure to this device has been a concern for different health professionals, as this behavior is being observed at an increasingly early age in children and adolescents.^5^ Among young Germans, around 92% of individuals between the ages of 12 and 19 reported using a smartphone daily.^6^ In Brazil, approximately 73.8% of 10-year-old children used the internet in 2019, with 98.6% of these individuals having a cell phone as their main form of access.^7^

In addition to the high prevalence of use of this device among children, the use considered dependent is also a negative aspect that can affect the health levels of the population in question. Problematic smartphone use (PSU) is characterized as excessive and problematic use of the device that can cause dependence, and can cause physical problems such as headaches, eye fatigue, back, shoulder, and neck pain, as well as situations of stress, depression, anxiety, and social deprivation, due to withdrawal from the device.^1,8^

Data on PSU and its consequences for the health of Brazilian adolescents show that individuals classified as dependent on smartphone use are more likely to have high levels of stress, anxiety, and depression.^9^ Furthermore, it is also evidenced in the literature that PSU can cause problems such as high amounts of sedentary behavior as well as poor sleeping habits.^2,10^

In order to monitor and assess PSU in young people, valid instruments for such observation have been reported in studies that address the topic in question. One of the instruments used in the literature in recent years is the Smartphone Addiction Scale-Short Version (SAS-SV),^11^ which was recently validated for university students in Brazil.^12,13^ This questionnaire contains 10 Likert-type questions and classifies the person being assessed as having a normal condition or a risk for PSU based on the sum of the points assigned to each question. In addition to Brazil, this instrument has also been translated and adapted for other countries such as Germany, Italy, Spain, and Turkey.^12^

However, when seeking to assess smartphone usage behaviors in children, this scenario is little explored in the literature, as there are currently no validated instruments to assess PSU in this population group. In this sense, validating competent instruments to assess PSU in children is necessary, mainly in order to verify the presence of this problem in the population in question as early as possible.

Thus, the objective of this article is to evaluate the validity, reproducibility, and reliability of the Brazilian version of the smartphone addiction scale in children aged 8–10 years.

## METHOD

This study is characterized as an intentional cross-sectional design. Data collection was carried out from August to December 2024. The data used in this article come from the research entitled: "Smartphone use in children: prevalence, associated factors and health consequences," which was approved by the Ethics and Research Committee of Federal University of Paraná under number: 60487322.5.0000.0102, as well as by the health ethics committee of the city of Curitiba, PR, under protocol: 07-067082/2022.

The sample of the present study consisted of children of both sexes, aged 8–10 years, regularly enrolled in three different schools of the municipal education network of the city of Curitiba, PR. The schools were intentionally selected based on their willingness to participate in the study. The students were recruited through convenience sampling. The minimum sample size was calculated using the G*Power 3.1.7 software, based on the t-test family, with an effect size of 0.30, alpha of 0.05, and statistical power of 0.95. An effect size of 0.30 represents a medium effect, which is commonly used in behavioral and health research to guide sample size estimation and to interpret the expected magnitude of the associations.

Inclusion criteria were: children aged 8–10 enrolled in participating schools, whose parents or legal guardians provided written informed consent, and who were able to participate in all study procedures. Exclusion criteria were: children with diagnosed cognitive disabilities that prevented them from understanding or responding to the questionnaires, children whose parents/guardians did not provide consent, and children whose questionnaires were completed incompletely. As a result, a minimum sample of 147 individuals was obtained.

In the first collection, 234 students were recruited. For the second collection, 201 students were evaluated; however, due to withdrawal from the study and incomplete completion of the questionnaire, the final sample was 194 students.

To begin data collection, the main researcher made a prior visit to the school with the intention of explaining the objectives of the research to the school administration. After obtaining permission from the school administration and obtaining the informed consent form (ICF) signed by the parents or guardians and assent forms by students, data collection was carried out at the school premises by trained researchers from the Center for Physical Activity and Health Studies at the Federal University of Paraná.

Initially, a pilot study was conducted with students aged 8–10 years old with the aim of testing the instruments that would be used. On the day of data collection, during regular class hours, after the teacher collected the ICF, the students answered the questionnaire related to sociodemographic data (sex, age, type of housing, type of residence, number of siblings, and father's and mother's education). The Brazil criterion^14^ was used with the intention of classifying children according to their socioeconomic class, with the following classification being used for the present study: A and B separately, and categories C, D, and E grouped together. For the parents’ education, the grouping was carried out into two categories: ≤8 years of study and >8 years of study.

Next, the questionnaire regarding smartphone addiction (SAS-SV) was applied. The original version of the SAS-SV is a validated self-report questionnaire developed to assess PSU and addictive behaviors in children and adolescents. It consists of 10 items, assessed on a 6-point Likert scale (1=strongly disagree to 6=strongly agree). Higher total scores indicate a greater tendency toward smartphone addiction. For the present study, the six possible answers were reduced to three to make it easier for the children to understand the questions, given the difficulty they presented during the pilot study. Regarding classification, the SAS-SV considers individuals with PSU to be those who present a score higher than 31 for boys, and higher than 33 for girls. A question was also created regarding whether the child owned their own smartphone. This question was answered exclusively by the parents or guardians when filling out the previously sent ICF.

For statistical treatment of the data, their normality was initially verified using the Kolmogorov-Smirnov test and histograms. The sample description was performed using absolute and relative frequencies. The chi-square test was used to observe possible associations between genders regarding categorical variables. Exploratory factor analysis was used to test the validity of the questionnaire, and items equal to or greater than 0.40 were considered to define the factors. In addition, the Kaiser-Meyer-Olkin (KMO) index and Bartlett's test of sphericity (BTS) were used to adapt the items for analysis, with KMO values ≥0.60 and BTS with statistical significance at the level of p<0.05 being considered satisfactory.

Regarding the temporal stability of the instrument, the reproducibility test (test–retest) was performed using the intraclass correlation coefficient (ICC), and after a 10-day interval, the students answered the questionnaire again in the classroom, with the same researcher present to administer the questionnaire. Reproducibility was considered adequate for ICC ≥0.70. For the analysis of the internal consistency of the scale, Cronbach's α was used, with alpha values ≥0.70 being considered adequate.^15^

All analyses were performed using SPSS software version 21.0, adopting a significance level of p<0.05.

## RESULTS

Table 1 shows the results regarding the sample description. The sample consisted of a total of 194 children, of which 89 (45.9%) were male and 105 (54.1%) were female. Most of the children had less than two siblings and lived with their father and mother, 148 (76.3%) and 123 (63.4%) respectively. The father was the head of the family in 61.5% of the total sample, and the most cited type of residence was house (53.6%). A total of 168 (90.3%) children had a mother with more than 8 years of schooling, and 148 (76.3%) reported having a father with more than 8 years of schooling. Regarding economic class, of the total sample, 15.8% were classified as A, 67.2% as B, and 16.9% as C/D/E. Regarding whether the child has a smartphone, 120 (64.9%) parents reported that their child has their own smartphone. Finally, 33 children (17%) were classified as having a PSU.

**Table 1:** Characteristics of the sociodemographic variables of the total sample and stratified by sex of schoolchildren aged 8–10 years from Curitiba, PR (n=194).

Variables		Total		Male		Female		χ^2^	p-value
		n	%	n	%	n	%		
Age (years)									
	8	52	26.8	25	28.1	27	25.7	0.51	0.474
	9	84	43.3	40	44.9	44	41.9		
	10	58	29.9	24	27.0	34	32.4		
Number of brothers									
	<2	148	76.3	75	84.3	73	69.5	5.79	**0.001** [Corresp c1]
	≥2	46	23.7	14	15.7	32	30.5		
Who do you live with?									
	Father	4	2.1	2	2.2	2	1.9	0.01	0.902
	Mother	41	21.1	20	22.5	21	20.0		
	Father and mother	123	63.4	53	59.6	70	66.7		
	Others	26	13.4	14	15.7	12	11.4		
Type of residence									
	House	104	53.6	50	56.2	54	51.4	2.19	0.534
	Townhouse	59	30.4	23	25.8	36	34.3		
	Apartment	28	14.4	15	16.9	13	12.4		
	Others	3	1.5	1	1.1	2	1.9		
Head of the family									
	Mother	74	38.5	40	45.5	34	32.7	3.27	0.070
	Father	118	61.5	48	54.5	70	67.3		
Mother's education (years old)									
	Under 8	18	9.7	8	9.5	10	9.8	0.00	0.950
	Over 8	168	90.3	76	90.5	92	90.2		
Father's education (years old)									
	Under 8	46	23.7	21	23.6	25	23.8	0.00	0.972
	Over 8	148	76.3	68	76.4	80	76.2		
Have a smartphone									
	Yes	120	64.9	56	65.1	64	64.6	0.00	0.952
	No	65	35.1	30	34.9	35	35.4		
Problematic smartphone use									
	Yes	33	17.0	17	19.1	16	89.0	0.50	0.474
	No	161	83.0	72	80.9	15	84.8		
Economy class									
	A	28	15.8	15	18.5	13	13.5	0.06	0.812
	B	119	67.2	51	63.0	68	70.8		
	C/D/E	30	16.9	15	18.5	15	15.6		

* p<0.01.

Bold indicates statistically significant p-values.

The results of the factor analysis (Table 2) showed the presence of three factors for the items of the PSU scale. The factors explained between 0.37 and 0.81 of the total variance of the scale. Factor 1 was composed of four items and had a factor loading ranging from 0.37 to 0.80. Regarding factor 2, it was composed of three items, and the variation of the factor loading was between 0.39 and 0.81. Finally, three items composed factor 3, where the variation of the factor loading was between 0.40 and 0.81. For the factor analysis procedures, the sample presented an adequate size (KMO>0.77; p<0.01 and Bartlett p<0.001).

**Table 2:** Exploratory factor analysis for the scale measuring smartphone dependence in children aged 8–10 in Curitiba (PR).

Scale			
Smartphone addiction questionnaire	Factor 1	Factor 2	Factor 3
Stopping planned tasks due to smartphone use	0.37		
Difficulty concentrating in class/homework due to smartphone use			0.81
Feeling pain in your wrists or neck while using your smartphone			0.70
There is nothing harder than being without your smartphone	0.77		
Getting impatient and irritated without your smartphone	0.80		
Thinking about your smartphone even when you're not with it	0.56		
Never stop using your smartphone, even though you know it causes problems and negative effects			0.40
Constantly using your smartphone to view social media posts		0.39	
Use your smartphone for longer than you intend to		0.75	
People around me say that I use my smartphone excessively		0.81	
% of variance explained	20.59	17.08	15.53
% of total variance explained	20.59	37.67	53.20
KMO	0.77		
Bartlett's test	<0.01		

KMO: Kaiser-Meyer-Olkin.

Table 3 shows the reproducibility (test-retest) and reliability (internal consistency) measures of the questionnaire. The test and retest results, obtained through the ICC, demonstrate that most items on the scale presented high agreement, with an overall score of 0.85. The only item that obtained a score lower than 0.50 was: "Difficulty concentrating in class or on homework due to the use of a smartphone," with a score of 0.34. The internal consistency analysis, performed through Cronbach's α, demonstrated that most items obtained a score higher than 0.50, with a total item score of 0.87.

**Table 3:** Measures of reproducibility (test-retest) and reliability (internal consistency) (n=194) — of the smartphone addiction questionnaire in children aged 8–10 years from Curitiba (PR).

Scales		
Smartphone addiction questionnaire	α	ICC (95%CI)
Stopping planned tasks due to smartphone use	0.57	0.55 (0.40–0.66)
Difficulty concentrating in class or on homework due to smartphone use	0.35	0.34 (0.12–0.50)
Feeling pain in your wrists or neck while using your smartphone	0.78	0.78 (0.70–0.83)
There is nothing harder than being without your smartphone	0.57	0.50 (0.34–0.62)
Getting impatient and irritated without your smartphone	0.63	0.63 (0.52–0.72)
Thinking about your smartphone even when you're not with it	0.68	0.67 (0.57–0.75)
Never stop using your smartphone, even though you know it causes problems and negative effects	0.67	0.65 (0.54–0.74)
Constantly checking your smartphone to access social media posts	0.74	0.73 (0.64–0.80)
Use your smartphone for longer than you intend to	0.81	0.81 (0.75–0.86)
People around me say that I use my smartphone excessively.	0.75	0.75 (0.67–0.81)
Overall score	0.87	0.85 (0.80–0.89)

α: Cronbach's alpha; ICC: intraclass correlation coefficient; 95%CI: 95% confidence interval.

## DISCUSSION

The prevalence data on smartphone use among children in this sample reveal that 17% of them were considered to have PSU. These findings are similar to those found in a sample of Swiss adolescents, where 16.9% were considered to have signs of smartphone addiction.^16^ Even though the samples are different in terms of age, it is observed that excessive use of smartphones, especially in the first decade of life, demonstrates a warning about the consequences that this use can cause for children over the years, as studies point to an association between prolonged use of smartphones and low academic performance, sleep disorders and increased sedentary behavior in adolescents.^6,10^

The PSU questionnaire presented moderate to satisfactory levels of validity, reproducibility, and reliability. These findings support the possibility of using this questionnaire in an assessment of smartphone use among children aged 8–10 years old, both sexes.

Satisfactory factor loadings were demonstrated for the questionnaire, with 53.2% of its total variance explained by three factors: the first was related to aspects of feeling, as it was composed of items that asked the respondent about what he or she feels when he or she is not using his or her device, with factor loadings ranging from 0.37 to 0.80; the second factor was related to the use of the device for a very long time, with factor loadings ranging from 0.39 to 0.81; and the third factor was related to the consequences of physical aspects resulting from smartphone use, with loadings ranging from 0.40 to 0.81. The presence of three factors was also found in a recent study carried out to validate the same instrument, however, among Japanese adults.^17^

The value of explained variance found in the factor analysis of the present study shows superiority when compared with the validation study of the same scale when carried out among Brazilian university students and adults (53.2×39.2%).^12^ Furthermore, the variance of the present study was similar to that of other studies that sought to validate the SAS-SV in other countries, such as Saudi Arabia, Spain, and Belgium.^18,19^ Thus, according to what is found in the literature in terms of validity, the SAS-SV can be a good instrument for assessing smartphone dependence in children aged 8–10. Furthermore, it is possible to state that the use of questionnaires, as already reported in the literature, is an effective form of assessment in epidemiological research, since it is a method in which it is possible to assess a large group of subjects at a low investment cost on the part of the researcher.^20,21^

Regarding the reliability values, obtained through Cronbach's α, the overall score of the present study was 0.87. When compared to the original study, carried out with adolescents from South Korea and with the intention of developing the short version of the SAS-SV, the Cronbach's α value is similar to that of the aforementioned study (0.87×0.91).^11^ In this sense, Cronbach's α values above 0.70 indicate good internal consistency of the questionnaire, suggesting that the interpretation of the questions by the respondents was satisfactory, making the instrument reliable.^22^

For reproducibility analysis, temporal testing of an instrument is an important procedure for verifying the consistency of responses given by participants at different times, thus presenting stable and replicable results.^23^ In this article, most of the items presented acceptable ICC values, with the overall score being 0.85. In a validation of the same instrument carried out among Japanese adults, the overall score was 0.70, which is lower than in the present study. The differences between the scores reported may be due to the interval used between the first and second application of the questionnaire. Unlike the interval used in the aforementioned study, that is, 7 months, a period of 10 days was used between one application and the next, and this protocol is in line with what is suggested when seeking to evaluate the temporal stability of a questionnaire, especially when it comes to evaluation among children.^15^

Still with the intention of ensuring adequate temporal stability for the scale evaluated in this research, the protocol adopted was the presence of the same researcher in the administration of the questionnaire at both times. Thus, it was possible to ensure greater solidity of the results, that is, to enable the instrument to reproduce very similar results even when administered at different times.^23^

Although this study can be considered a step forward in validating an instrument for assessing smartphone use in children, it has limitations that should be highlighted: a sample was intentionally recruited and not representative, limiting the extrapolation of these results; the children participating in this study are from a specific city in southern Brazil, which may compromise the results in terms of behavior and cultural aspects when compared to Brazilian children from other regions. It is also noteworthy that the sample came exclusively from public schools, which makes it impossible to extrapolate results to students in private schools. Furthermore, due to the lack of an instrument classified as a reference standard for assessing smartphone use in children, the comparison of this measurement scale with a standard instrument is limited. Therefore, it is necessary to encourage the production of new studies that will fill those gaps, thus contributing to a better understanding of the consequences that smartphone use can have on children's health.

In addition, the scale validated in this article is presented as a novelty to assess PSU in children. Thus, this instrument can help researchers and professionals in the area to better understand the consequences that prolonged smartphone use can cause to children's health. It is also noteworthy that understanding this behavior can promote future interventions with the intention that these electronic devices, such as smartphones, can be used in a healthy and balanced way among children.

In conclusion, the SAS-SV presents adequate validity, reproducibility, and reliability to assess smartphone use in children aged 8–10 in public schools.

## Data Availability

The database that originated the article is available with the corresponding author. CAAE: 60487322.5.0000.0102.
